# Clonal Spread of *Escherichia coli* ST93 Carrying *mcr-1*-Harboring IncN1-IncHI2/ST3 Plasmid Among Companion Animals, China

**DOI:** 10.3389/fmicb.2018.02989

**Published:** 2018-12-04

**Authors:** Jing Wang, Xin-Yi Huang, Ying-Bi Xia, Ze-Wen Guo, Zhen-Bao Ma, Meng-Ying Yi, Lu-Chao Lv, Pei-Lan Lu, Jie-Cong Yan, Jia-Wei Huang, Zhen-Ling Zeng, Jian-Hua Liu

**Affiliations:** College of Veterinary Medicine, Key Laboratory of Zoonosis of Ministry of Agricultural and Rural Affairs, South China Agricultural University, Guangzhou, China

**Keywords:** colistin resistance, companion animals, *Escherichia coli*, *mcr-1*, plasmids

## Abstract

The purpose of this study was to investigate the occurrence of plasmid-mediated colistin resistance gene *mcr-1* in Enterobacteriaceae isolates from companion animals in Guangzhou, China. Enterobacteriaceae isolated from 180 samples collected from cats and dogs were screened for *mcr-1* by PCR and sequencing. MCR-1-producing isolates were further characterized by multilocus sequence typing and pulsed-field gel electrophoresis (PFGE). Plasmid characterization was performed by conjugation, replicon typing, S1-PFGE, and Southern blot hybridization. Plasmid pHN6DS2 as a representative IncN1-IncHI2/ST3 plasmid from ST93 *E. coli* was fully sequenced. pHN6DS2-like plasmids were screened by PCR-mapping and sequencing. The *mcr-1* gene was detected in 6.25% (8/128) *Escherichia coli* isolates, of which, five belonged to *E. coli* ST93 and had identical PFGE patterns, resistance profiles and resistance genes. *mcr-1* genes were located on ∼244.4 kb plasmids (*n* = 6), ∼70 kb plasmids, and ∼60 kb plasmids, respectively. Among them, five *mcr-1*-carrying plasmids were successfully transferred to recipient by conjugation experiments, and were classified as IncN1-IncHI2/ST3 (∼244.4 kb, *n* = 4, all obtained from *E. coli* ST93), and IncI2 (∼70 kb, *n* = 1), respectively. Plasmid pHN6DS2 contained a typical IncHI2-type backbone, with IncN1 segment (Δ*repA-*Iterons I-*gshB*-ΔIS*1294*) inserted into the multiresistance region, and was similar to other *mcr-1*-carrying IncHI2/ST3 plasmids from Enterobacteriaceae isolates of various origins in China. The remaining five *mcr-1*-bearing plasmids with sizes of ∼244.4 kb were identified to be pHN6DS2-like plasmids. In conclusion, clonal spread of ST93 *E. coli* isolates was occurred in companion animals in Guangzhou, China.

## Introduction

Colistin has been a last-resort treatment option in human medicine for infections caused by multi-resistant Gram-negative bacteria (Kaye et al., 2016). Resistance to colistin had only been involved with chromosomal mutations until the identification of plasmid-mediated colistin resistance gene *mcr-1* from a porcine *Escherichia coli* isolate in China in 2015 (Liu et al., 2016). The emergence and dissemination of *mcr-1* is a significant global concern and poses a serious threat to clinical treatment. Since the discovery of *mcr-1*, it has been increasingly reported in Enterobacteriaceae from humans, animals, food products, and the environment worldwide, particularly in China (Jeannot et al., 2017; Wang et al., 2018). To date, *mcr-1* has been identified on various plasmid types, with IncI2, IncHI2, and IncX4 being the major carriers, and IS*Apl1* is involved in *mcr-1* mobilization between DNA molecules (e.g., plasmid, chromosome) (Matamoros et al., 2017; Li et al., 2018; Wang et al., 2018). Previous studies have demonstrated high *mcr-1* prevalence in *E. coli* isolates from food-producing animals (pigs and chickens) and meat (pork and chicken meat) in China (Liu et al., 2016, 2017; Wu et al., 2018). However, *mcr-1* has been rarely reported in companion animals, though 8.7% Enterobacteriaceae isolates were identified to carry *mcr-1* among companion animals in Beijing, China, meanwhile, *mcr-1*-positive *E. coli* isolates may transfer between companion animals and close contactors in a pet store in Guangzhou, China (Sun et al., 2016; Zhang et al., 2016; Lei et al., 2017). Thus, in this study, we investigated the prevalence and characterization of *mcr-1* in Enterobacteriaceae isolates from companion animals in Guangzhou, China, to provide insights into the spread of *mcr-1* in companion animals.

## Materials and Methods

### Sample Collection and *mcr-1* Detection

During July to August 2016, 180 samples were collected from cats and dogs at four animal hospitals located in four districts in Guangzhou, China, including 68 feces samples from healthy animals, 112 samples (feces, urine, eye secretion, ear exudates, nasal secretion, and skin) from diseased animals (Table 1). Samples were incubated in LB broth for 16∼24 h and then inoculated on the MacConkey agar. One isolate per sample was selected and identified by MALDI-TOF mass spectrometry or 16S rRNA sequencing (Supplementary Table S1). In all isolates, *mcr-1* was detected by PCR and sequencing (Supplementary Table S1).

**Table 1:** Source and origin of Enterobacteriaceae isolates obtained from companion animals in Guangzhou, China.

Sampling Location	Sampling time	Specimen source	No. of samples	Sick or healthy	Specimen type (no. of samples)	No. of isolates		
						*E. coli K.pneumoniae E. cloacae*		
Animal hospital 1	Aug 5th–12th	cat	2	S	Feces (1) Urine (1)	1		
		dog	28	S	Feces (14) Urine (14)	14	8	2
		All	30			15		
Animal hospital 2	July 10th–17th	cat	15	H	Feces (3)	2		
	July 26th–Aug 3rd			S	Feces (10), urine (1), eye secretion (1)	10		1
		dog	36	H	Feces (30)	25	4	
				S	Feces (3), urine (3)	4		
		All	51			41		
Animal hospital 3	July 12th–19th	cat	12	H	Feces (5)	5		
	July 21st–30th			S	Feces (4), urine (1), eye secretion (1), ear exudates (1)	2		
		dog	43	H	Feces (15)	11		1
				S	Feces (15), urine (1), nasal secretion (8), ear exudates (3), skin (1)	16	5	
		All	55			34		
Animal hospital 4	Aug 13th–20th	cat	6	H	Feces (4)	4		
				S	Feces (2)	1		
		dog	38	H	Feces (11)	9	1	
				S	Feces (27)	24	3	
		All	44			38		
Total			180			128	21	4


*H, healthy companion animals for vaccination; S, sick companion animals diagnosed by veterinarians, including sick animals with hematuresis, urethritis, pneumonia, diarrhea, cough, bone fracture, patellar dislocation, tumor, hysterectomy, otitis externa, canine distemper, sarcoptic acariasis, cystitis, hepatitis, hydrocephalus, and some unknown diseases. Samples were consecutively taken from all animals admitting the four hospitals during sampling time.*

### Molecular Typing

The genetic diversity of *mcr-1*-positive *E. coli* isolates was characterized by multilocus sequence typing (MLST)^1^. Five *mcr-1*-carrying ST93 *E. coli* isolates in this study and the *mcr-1*-positive ST93 *E. coli* strain PET01, that was previously obtained from a cat in Guangzhou, China (Zhang et al., 2016) were further analyzed by pulsed-field gel electrophoresis (PFGE) (Gautom, 1997).

### Conjugation/Transformation Experiments and Plasmid Characterization

Conjugation experiments were carried out using streptomycin-resistant *E. coli* C600 as the recipient strain as previously described (Chen et al., 2007). Transconjugants were selected using 2 mg/L colistin and 3,000 mg/L streptomycin. Transfer frequencies were calculated as the number of transconjugants per recipient, experiments were performed in triplicate. Transformation was conducted by heat-shock and electroporation using *E. coli* strain DH5α as the recipient strain, and selected by 2 mg/L colistin. The presence of *mcr-1* in the transconjugants was confirmed by PCR and sequencing. Transconjugants with a single *mcr-1*-carrying plasmid, verified by S1-PFGE (Barton et al., 1995) and Southern blot hybridization, were selected for further study. The location of *mcr-1* in the original isolates which failed to obtain transconjugants/transformants was determined by S1-PFGE and Southern blot hybridization. All the transconjugants were characterized by PCR-based replicon typing and IncI2 and IncX4 plasmids were screened according to previously described protocols (Carattoli et al., 2005; Johnson et al., 2012; Lv et al., 2013). IncHI2 plasmids were further characterized by plasmid double locus sequence typing (García-Fernández and Carattoli, 2010). The genetic structure of *mcr-1* was determined by PCR mapping and sequencing in five transconjugants and three original isolates without transconjugants/transformants (Supplementary Table S2).

### Antimicrobial Susceptibility Testing

The original *mcr-1*-positive *E. coli* isolates, the recipient strain C600, and transconjugants were tested for their susceptibility to ampicillin, cefotaxime, imipenem, gentamycin, amikacin, tetracycline, chloramphenicol, florfenicol, ciprofloxacin, sulfamethoxazole/trimethoprim, colistin, and fosfomycin by the agar dilution method or the broth microdilution method (limited to colistin). Antimicrobial susceptibility tests were performed and interpreted according to M100, 28th edition of the CLSI (Wayne, PA, United States) (Clinical Laboratory Standards Institute [CLSI], 2018). Colistin ( > 2 mg/L), and florfenicol ( > 16 mg/L) were interpreted according to the clinical breakpoints or epidemiological cutoff values of EUCAST.^2^ The *E. coli* strain ATCC 25922 was used for quality control. The mutations within *gyrA* and *parC* were detected in ciprofloxacin-resistant *mcr-1*-positive *E. coli* isolates (Supplementary Table S1). Other resistance genes, including *bla*_CTX-M_, *floR*, *rmtB*, *oqxAB*, and *fosA3* were screened in original *mcr-1*-positive isolates and their transconjugants using the primers listed in Supplementary Table S1.

### Plasmid Sequencing

Plasmid pHN6DS2, as a representative IncN1-IncHI2/ST3 plasmid from ST93 *E. coli* isolate, was selected to extract from the transconjugant using QIAGEN^®^ Plasmid Midi Kit (Qiagen, Hilden, Germany) and sequenced by Illumina Miseq technology (Illumina, San Diego, CA, United States). Sequence reads were assembled into contigs with SOAPdenovo version 2.04. Nine contigs of pHN6DS2 were assembled into the complete plasmid sequence with PCR amplification and Sanger sequencing (Supplementary Table S3) using related *mcr-1*-carrying plasmids as references by BLAST^3^. Analysis and annotation of plasmid pHN6DS2 were performed using the RAST server (Aziz et al., 2008), ISfinder^4^, ResFinder^5^, RAC^6^, BLAST^7^, and the Gene Construction Kit 4.5 (Textco BioSoftware, Inc., Raleigh, NC, United States). The remaining transconjugants or original isolates containing ∼244.4 kb *mcr-1*-bearing plasmid were examined for pHN6DS2-like plasmids by PCR and sequencing (Supplementary Table S4).

### Nucleotide Sequence Accession Number

The nucleotide sequences of plasmid pHN6DS2 has been deposited in the GenBank database under the accession number MH459020.

## Results and Discussion

### Identification of *mcr-1* and Antimicrobial Susceptibility

A total of 128 *E. coli*, 21 *Klebsiella pneumoniae*, and 4 *Enterobacter cloacae* isolates were obtained from 180 samples of companion animals origin (Table 1). Among them, *mcr-1* was present in eight (6.25%) *E. coli* isolates, two from healthy animals and six from diseased animals (Table 2). The isolates from diseased animals (6/72, 8.33%) showed higher *mcr-1* prevalence than those from healthy animals (2/56, 3.57%; *P* > 0.05). However, we did not identify *mcr-1* in *K. pneumoniae* or *E. cloacae* isolates. Although *mcr-1* prevalence in companion animals was greatly lower than that among food-producing animals in China (Liu et al., 2017; Wu et al., 2018), it was similar to the previously described *mcr-1* detection in companion animals in Beijing, China (Lei et al., 2017).

**Table 2:** Characteristics of *mcr-1*-carrying *E. coli* isolates.

Strain	Origin (Physical condition)	Sampling location and time	MLST (ST)	Other resistance genes	Colistin MIC (mg/L)	mutations		Other resistance patterns	Genetic structure of *mcr-1*	Location of *mcr-1* (plasmid)
						*gyrA*	*parC*			
GZ6DS2^∗^	dog-1, urine (hematuresis)	Hospital 2, July 2016	93	*bla*_CTX-M-64_/ *bla*_CTX-M-14_/*floR*/*fosA3*	4	S83L D87Y	S57T S80I	AMP/CTX/GEN/TET/ CHL/FFC/SXT/FOS/CIP	IS*Apl1*-*mcr-1*-pap2	∼244.4 kb IncN1-IncHI2/ST3
GZ6DS9^∗^	dog-2, nasal secretion (pneumonia)	Hospital 3, July 2016	93	*bla*_CTX-M-64_/ *bla*_CTX-M-14_/*floR*/*fosA3*	4	S83L D87Y	S57T S80I	AMP/CTX/GEN/TET/ CHL/FFC/SXT/FOS/CIP	IS*Apl1*-*mcr-1*-pap2	∼244.4 kb IncN1-IncHI2/ST3
GZ6CS9	cat-1, feces (diarrhea)	Hospital 2, July 2016	93	*bla*_CTX-M-64_/ *bla*_CTX-M-14_/*floR*/*fosA3*	4	S83L D87Y	S57T S80I	AMP/CTX/GEN/TET/ CHL/FFC/SXT/ FOS/CIP	IS*Apl1*-*mcr-1*-pap2	∼244.4 kb
GZ6DH17^∗^	dog-3, feces (H)	Hospital 3, July 2016	93	*bla*_CTX-M-64_/ *bla*_CTX-M-14_/*floR*/*fosA3*	4	S83L D87Y	S57T S80I	AMP/CTX/GEN/TET/ CHL/FFC/SXT/ FOS/CIP	IS*Apl1*-*mcr-1*-pap2	∼244.4 kb IncN1-IncHI2/ST3
GZ6DH18^∗^	dog-4, feces (H)	Hospital 3, July 2016	93	bla_CTX-M-64_/ bla_CTX-M-14/floR_/fosA3	4	S83L D87Y	S57T S80I	AMP/CTX/GEN/TET/ CHL/FFC/SXT/ FOS/CIP	ISApl1-mcr-1-pap2	∼244.4 kb IncN1-IncHI2/ST3
GZ6DS4^∗^	dog-5, feces (cough, diarrhea)	Hospital 2, July 2016	1011	bla_CTX-M-64_/floR/ fosA3/oqxAB	4	S83L D87Y	S80I	AMP/CTX/GEN/TET/ CHL/FFC/SXT/FOS/CIP	mcr-1-pap2	∼70 kb IncI2
GZ6DS68	dog-6, feces (S)	Hospital 4, Aug 2016	3285	bla_CTX-M-55_/fosA3	4	S83L D87Y	S80I	AMP/CTX/TET/SXT/ FOS/CIP	mcr-1-pap2	∼60 kb
GZ6DS69	dog-7, feces (S)	Hospital 4, Aug 2016	NEW	bla_CTX-M-14_/ bla_CTX-M-15_/floR/ fosA3/oqxAB	4			AMP/CTX/GEN/TET/ CHL/FFC/SXT/FOS	ISApl1-mcr-1-pap2	∼244.4 kb


*AMP, ampicillin; CTX, cefotaxime; GEN, gentamycin; TET, tetracycline; CHL, chloramphenicol; FFC, florfenicol; SXT, sulfamethoxazole/trimethoprim; FOS, fosfomycin; CIP, ciprofloxacin. All isolates were susceptible to amikacin and imipenem. Asterisk indicates isolates from which the mcr-1 gene can be transferred to recipients by conjugation experiments. H, healthy; S, sick, specific disease was not recorded or unknown. The isolate was identified as New ST with alleles adk10, fumC27, gyrB4, icd10, mdh8, purA1, and recA2, respectively. The most related ST is ST7507 with alleles adk10, fumC27, gyrB4, icd10, mdh8, purA8, and recA2, respectively. Resistance genes and resistance phenotypes transferred to the recipient by conjugation experiments are underlined.*

As shown in Table 1, all *mcr-1*-positive strains exhibited minimal inhibitory concentration (MIC) of 4 mg/L to colistin, and showed resistance to ampicillin, tetracycline, sulfamethoxazole/trimethoprim, and fosfomycin, but susceptibility to amikacin and imipenem; seven displayed resistance to gentamycin, chloramphenicol and florfenicol. The *mcr-1*-positive isolates also harbored other resistance genes, including *bla*_CTX-M_ (*n* = 8), *fosA3* (*n* = 8), *floR* (*n* = 7), and *oqxAB* (*n* = 2) (Table 2). In addition, seven *mcr-1*-bearing isolates exhibited resistance to ciprofloxacin with mutations in *gyrA* (S83L and D87Y) and *parC* (S57T and/or S80I) (Table 2).

Since colistin is not applied to companion animals in China, pet food containing chicken meat might be one source of *mcr-1* (Lei et al., 2017). Close contact to food-producing animals in local hog and poultry markets, as well as to humans, might also be the potential origins. Furthermore, the widely use of cephalosporins, aminoglycosides, and fluoroquinolones in companion animal medicine (data not shown) could allow for the co-selection of isolates harboring *mcr-1*, as well as *bla*_CTX-M_ and *fosA3*, conferring resistance to crucial clinical antibiotics.

### Molecular Typing

**FIGURE 1:**
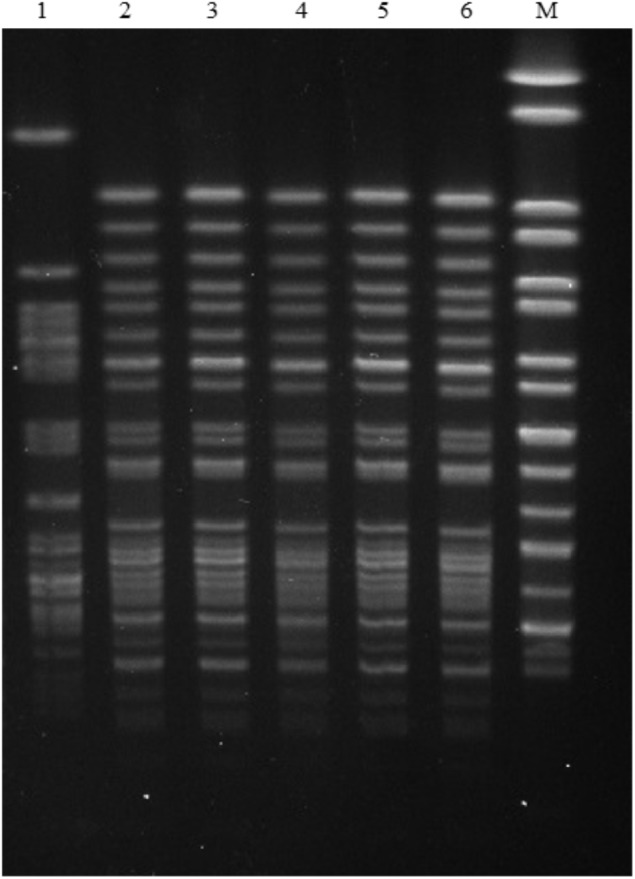
PFGE patterns of five *mcr-1*-carrying *E. coli* ST93 strains in this study and the *mcr-1*- carrying *E. coli* ST93 strain PET01 that was isolated from a cat in a pet shop from Guangzhou (Zhang et al., 2016). Lanes: (1) PET01; (2) GZ6DS2; (3) GZ6DS9; (4) GZ6CS9; (5) GZ6DH17; (6) GZ6DH18; (9) GZ6DS69; (10) GZ6DS68; M *Salmonella enterica* serovar Braenderup H9812 Marker.

Eight *mcr-1*-positive *E. coli* isolates were assigned to ST93 (*n* = 5), ST1011, ST3285, and a new ST, respectively (Table 2). ST93 has been sporadically described as avian and human extra-intestinal pathogenic or diarrhoeagenic *E. coli* in humans, animals, and food products worldwide (Chen et al., 2014; Maluta et al., 2014; Vogt et al., 2014), and particularly it has been previously detected as *mcr-1* carriers from a pig in Laos (Olaitan et al., 2015, 2016), from a cat in Guangzhou, China (Zhang et al., 2016), and from a patient in Finland (Gröndahl-Yli-Hannuksela et al., 2018). The five *mcr-1*-carrying ST93 *E. coli* isolates were obtained from both intestinal and extraintestinal sites from two animal hospitals located within a distance of 7 km. They showed indistinguishable PFGE patterns which differed from previously described *mcr-1*-harboring ST93 *E. coli* isolate PET01 from a cat in Guangzhou (Zhang et al., 2016; Figure 1), indicating that clonal spread of *mcr-1*-harboring *E. coli* had occurred among companion animals within two hospitals in Guangzhou. The observation that they had identical antimicrobial susceptibility profiles, resistance genes, and mutations within *gyrA* and *parC* may further support this hypothesis (Table 2). However, small numbers of samples were collected from four animal hospitals in this study, thus limiting this hypothesis. The prevalence and dissemination mechanisms of *mcr-1* in companion animals in Guangzhou should be further investigated by using large scale samples from more animal hospitals. Though rare, it is possible for these two hospitals to exchange animal patients. The possibility of acquisition of *mcr-1*-harboring ST93 *E. coli* from a common ancestor could not be ruled out. Although horizontal transfer mediated by mobile elements such as insertion sequence and plasmids has been the major reason for *mcr-1* worldwide dissemination, clonal spread of *mcr-1*-harboring strains, such as *E. coli* ST93 in the present study, *Salmonella* Typhimurium ST34 in pigs (Li et al., 2016; Yi et al., 2017) might be another reason accounting for *mcr-1* transmission. Most importantly, the potential of *mcr-1* transmission mediated by MCR-1-producing clones from companion animals to humans through close contact should not be underestimated, which might have already occurred in China by *E. coli* ST354 and ST101 clones (Zhang et al., 2016; Lei et al., 2017).

### Characterization of *mcr-1*-Carrying Plasmids

Five strains successfully transferred *mcr-1* to *E. coli* C600 at frequencies of 10^-2^ to 10^-4^ transconjugants/recipient (Supplementary Table S5), and the remaining three strains failed to transfer *mcr-1* to *E. coli* C600 or DH5α by conjugation or transformation. S1-PFGE and Southern hybridization indicated that *mcr-1* was located on ∼244.4 kb plasmids (*n* = 6), ∼60 kb plasmids (*n* = 1), or ∼70 kb plasmid (*n* = 1) (Table 2). Additionally, five *mcr-1*-harboring transconjugants with single plasmid were classified as IncN1-IncHI2/ST3 (∼244.4 kb, *n* = 4) which were all obtained from ST93 *E. coli* isolates, and IncI2 (∼70 kb, *n* = 1) (Table 2 and Supplementary Figure S1), which agree with previous observation that IncHI2 and IncI2 plasmids have been the major vectors for *mcr-1* global dissemination (Matamoros et al., 2017; Wang et al., 2018). Furthermore, the transconjugants showed elevated MICs for colistin (1∼2 mg/L; 8-16-fold) compared with the recipient *E. coli* C600. In addition, co-transfer of resistance to ampicillin, cefotaxime, gentamycin, chloramphenicol, florfenciol, sulfamethoxazole/trimethoprim, and fosfomycin was observed in four transconjugants with IncN1-IncHI2/ST3 plasmid from ST93 *E. coli* isolates, resistance genes *bla*_CTX-M-14_, *floR* and *fosA3* were also co-transferred with *mcr-1* (Table 2). The co-transfer of *bla*_CTX-M-64_ with *mcr-1* on an IncI2 plasmid in the remaining transconjugant caused resistance to ampicillin and cefotaxime (Table 2). The presence of other resistance genes co-located on the same plasmid allows for the selection of *mcr-1* under pressure posed by other agents, thus facilitating *mcr-1* transmission.

It has been hypothesized that *mcr-1* is initially captured and mobilized by the composite transposon Tn*6330* (IS*Apl1*-*mcr-1*-*pap2*-IS*Apl1*), followed by the loss of IS*Apl1* over time, leading to the formation of *mcr-1* in diverse genetic structures, with the structure *mcr-1-pap2* being dominant, followed by the structure IS*Apl1*-*mcr-1*-*pap2* (Snesrud et al., 2018; Wang et al., 2018). The genetic structure of *mcr-1* in our study was determined by PCR mapping. We did not observe the complete Tn*6330*, but the presence of IS*Apl1* upstream was common, identified in six transconjugants or original isolates with ∼244.4 kb *mcr-1*-carrying plasmids, the structure *mcr-1*-*pap2* was also identified (*n* = 2) (Table 2). Our results further support that mobile elements (IS*Apl1*, IncHI2, and IncI2 plasmids) play an important role in the mobilization and dissemination of *mcr-1* in *E. coli* from different sources.

### Plasmid Sequencing and Comparative Analysis

Plasmid pHN6DS2 had a size of 253, 783 bp, and was organized similarly to other IncHI2 plasmids, containing regions for functions of replication, multiresistance, conjugal transfer, maintenance, and stability (Supplementary Figure S2).

**FIGURE 2:**
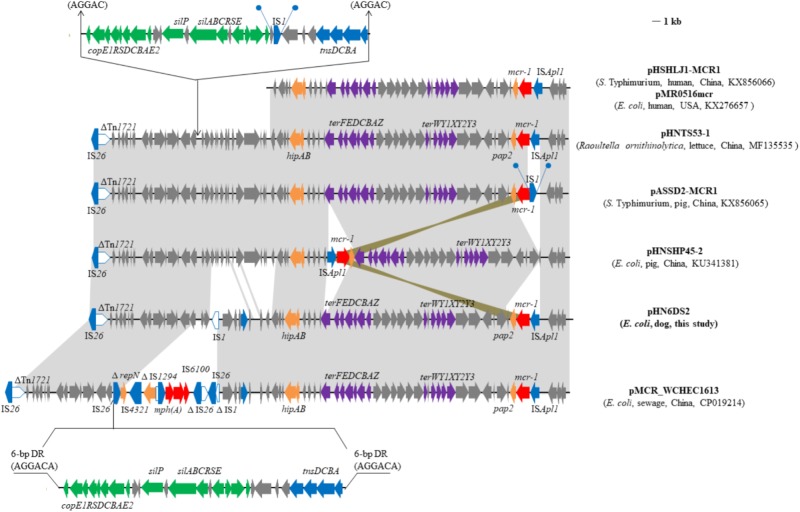
Genetic organization of the partial sequence containing *mcr-1* of plasmid pHN6DS2, and structural comparison with other *mcr-1*-carrying plasmids. Arrows indicate the positions of the genes and the direction. Regions with > 99% homology are shaded in gray or azure. Δ indicates a truncated gene or mobile element. Direct repeats are indicated by arrows and blue circles. Tall bars represent the 38 bp inverted repeat (IR) of transposons. The backbone is indicated by dotted lines.

Interestingly, a fragment with the least size of 37, 258-bp including the module. IS*Apl1*-*mcr-1*-*pap2* and a set of tellurite resistance determinants (*ter*YXWZABCDEF) in plasmid pHN6DS2 from canine *E. coli* was similar to several other IncHI2/ST3 plasmids found in Enterobacteriaceae isolates from various sources in China, such as plasmids pHNTS53-1 (*Raoultella ornithinolytica*, lettuce, MF135535), pHSHLJ1-MCR1 (*S.* Typhimurium, human, KX856066), pMCR_WCHEC1613 (*E. coli*, environment, CP019214) (Zhao et al., 2017), and pASSD2-MCR1 (*S.* Typhimurium, pig, KX856065) (Figure 2). However, IS*Apl1* was absent and IS*1* was inserted upstream of *mcr-1* flanked by 9-bp direct repeats (DRs) in plasmid pASSD2-MCR1 (Figure 2). Furthermore, the fragment was also identical to the corresponding region of the IncHI2-IncF recombinant plasmid pMR0516mcr obtained from clinical *E. coli* isolate in the United States (McGann et al., 2016; Figure 2). However, the module IS*Apl1*-*mcr-1*-*pap2* was inserted into the backbone of plasmid pHNSHP45-2 (Zhi et al., 2016) with different location and orientation (Figure 2).

**FIGURE 3:**
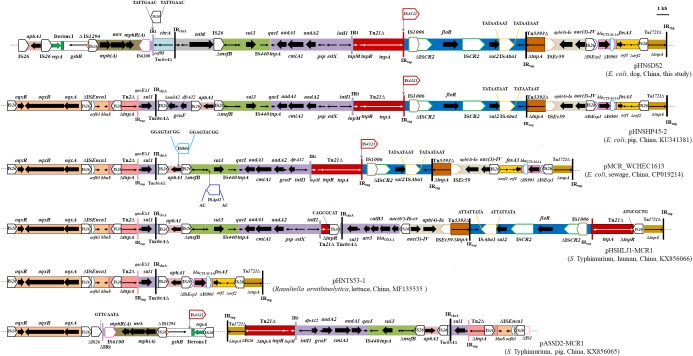
Genetic organization of the multiresistance region of plasmid pHN6DS2, and structural comparison with other *mcr-1*-carrying plasmids. The extents and directions of antibiotic resistance (thick arrows) and other genes are indicated. Δ indicates a truncated gene or mobile element. ISs are shown as boxes labeled with their name. Labeled vertical arrows with IS boxes indicate the insertion sites of IS elements. Direct repeats are indicated by arrows and sequence. Tall bars represent the 38 bp inverted repeat (IR) of transposons. The backbone is indicated by dotted lines.

Furthermore, the multiresistance region (MRR) of pHN6DS2 contained numerous resistance genes, such as *aphA1*, *tetM*, *sul3*, *aadA1*, *cmlA1*, *aadA2*, *floR*, *bla*_CTX-M-14_, and *fosA3*, and complete or truncated insertion sequences and transposons (e.g., IS*26*, Tn*21*, IS*4321*, IS*1006*, IS*CR2*, IS*Aba1*, Tn*5393*, IS*Ec59*, IS*Ecp1*, IS*903*, and Tn*1721*) (Figure 3). As a multi-replicon plasmid, pHN6DS2 harbored an approximately 3-kb IncN1 segment (Δ*repA-*Iterons I-*gshB*-ΔIS*1294*), containing IncN replication initiation gene *repA* truncated by IS*26*, five tandem 37-bp repeats within iterons region, *gshB* encoding glutathione synthetase, and 114 bp of the *ori*IS end of IS*1294* (Figure 3). The similar structure was also observed in plasmid pASSD2-MCR1 with the exception of IS*4321* insertion (Figure 3). The macrolide phosphotransferase region harboring several *mph* genes was located downstream of the IncN segment, and was followed by IS*6100*, 123-bp of Tn*402*, incomplete Tn*21*-like transposon Tn*chrA*, and a 2, 067-bp segment containing tetracycline resistance gene *tetM* (Figure 3). IS*26* was inserted in inverted repeat at the *tni* end of Tn*402*, named IRt, flanked by 8-bp DRs (Figure 3).

The MRR of pHN6DS2 was similar to that of pHNSHP45-2, but differed by acquisition of the ∼10.5-kb *oqxAB* resistance module (Tn*6010*-ΔIS*Enca1*-*orf63*-*blmS*-ΔTn*2*-IS*26*-ΔTn*2*) and an ∼4.3-kb segment harboring the |*qacEΔ1*|*sul1*| Δ*aadA2*|*gcuF*|*dfrA12*| cassette array which was interrupted by partial Tn*chrA* and IS*26*, and by loss of the IncN segment, *mph* region, and *tetM* region (Figure 3). Similarly, MRRs of *mcr-1*-carrying plasmids pMCR_WCHEC1613, pHSHLJ1-MCR1, pHNTS53-1, and pASSD2-MCR1 were related to those of pHN6DS2 and pHNSHP45-2, differed by insertions, deletions, or rearrangement of various regions harboring antimicrobial resistance genes such as *oqxAB*, *sul3*, *floR*, *bla*_CTX-M-14_, and *fosA3*, and mobile element such as Tn*21*, IS*CR2*, IS*4321* (Figure 3). Notably, IS*26*, via transposition and homologous recombination, seems to play an important role in the formation of distinct but also related MRRs. The remaining three transconjugants carrying IncN1-IncHI2/ST3 plasmids and two original isolates with ∼244.4 kb *mcr-1*-bearing plasmids were also examined for pHN6DS2-like plasmids. All five transconjugants or original isolates harbored pHN6DS2-like plasmids with identical insertion of the module IS*Apl1*-*mcr-1*-*pap2* but variable MRRs (Table 3).

**Table 3:** Characteristics of pHN6DS2-like plasmids in this study.

Isolates	HP1-IS*26*-*aphA1*	IS*26*-*repN*	*aphA1*-IS*26*-*repN*	*gshB*-IS*1294*-IS*26*-*mphA*	*fosA3*-IS*26*-Tn*1721*	Tn*1721*-HP2	IS*Apl1*-*mcr-1*-*pap2* insertion site at plasmid
GZ6DS9-2C^∗^	P	P	P	N	P	P	Like pHN6DS2
GZ6DH17-3C^∗^	P	P	N	N	P	P	Like pHN6DS2
GZ6DH18-1C^∗^	P	P	N	N	P	P	Like pHN6DS2
GZ6CS9	P	P	P	N	P	P	Like pHN6DS2
GZ6DS69	N	P	N	P	P	P	Like pHN6DS2


*Asterisk indicates transconjugants; HP: hypothetical protein, located in IncHI2 plasmid backbone. P, positive; N, negative.*

These data suggested that similar *mcr-1*-carrying IncHI2/ST3 plasmids, after acquiring, losing or reorganizing various regions, could spread among Enterobacteriaceae species in livestock, humans, vegetables, and the environment, particularly in different regions in China. The presence of pHN6DS2-like plasmids further supported this hypothesis and highlighted the potential of pHN6DS2-like plasmid to become an efficient vehicle for *mcr-1* dissemination between distinct organisms or regions.

## Conclusion

In conclusion, the spread of *mcr-1* in companion animals in the present study might be mainly attributed to clonal dissemination of *E. coli* ST93 isolates within two hospitals, associated with IncN1-IncHI2/ST3 plasmids. Although the origin of *mcr-1* in companion animals is unclear, it is possible for *mcr-1*-positive clones or plasmids to transfer from companion animals to humans through close contact, thus the dissemination of *mcr-1* among companion animals needs continued vigilance.

## Ethics Statement

This study was carried out in accordance with the recommendation of ethical guidelines of South China Agricultural University. Individual written informed consent for the use of samples was obtained from all the animal owners.

## Author Contributions

J-HL, Z-LZ, and JW conceived the study. X-YH, Y-BX, JW, Z-WG, Z-BM, M-YY, L-CL, P-LL, J-CY, and J-WH carried out the experiments. JW, X-YH, and Y-BX analyzed the data. JW wrote the manuscript. J-HL and Z-LZ revised the manuscript. All authors read and approved the final manuscript.

## Conflict of Interest Statement

The authors declare that the research was conducted in the absence of any commercial or financial relationships that could be construed as a potential conflict of interest.

## Footnotes

1 http://enterobase.warwick.ac.uk/species/index/ecoli

2 https://mic.eucast.org/Eucast2/

3 http://blast.ncbi.nlm.nih.gov/Blast.cgi

4 https://www-is.biotoul.fr//

5 https://cge.cbs.dtu.dk//services/ResFinder/

6 https://galileoamr.arcbio.com/mara/

7 http://blast.ncbi.nlm.nih.gov/Blast.cgi
