# Supplementary material for: Clonal Spread of Escherichia coli ST93 Carrying mcr-1-Harboring IncN1-IncHI2/ST3 Plasmid Among Companion Animals, China
Source: Front Microbiol. 2018 Dec 4;9:2989. doi: 10.3389/fmicb.2018.02989 (PMC6288184; doi:10.3389/fmicb.2018.02989)
Supplement: Supplementary file 1 [file Data_Sheet_1.PDF]

Table S1 Primers used for PCR and DNA sequencing in this study

Gene	Primer name	Sequence (5' to 3')	Size (bp)	Reference
mcr-1	mcr-F	TCGCGGCATTCGTTATA	582	This study
	mcr-R	GGTGGCGTTCAGCAGTC		This study
bla$_{\text{CTX-M-1 group}}$	M1-F	CTTCCAGAATAAGGAATCCC	949	1
	M1-R	CGTCTAAGGCGATAAACAAA		1
bla$_{\text{CTX-M-9 group}}$	M9-F	TGACCGTATTGGGAGTTTG	902	1
	M9-R	ACCAGTTACAGCCCTTCG		1
fosA3	fosA3-F	GCGTCAAGCCTGGCATTT	282	2
	fosA3-R	GCCGTCAGGGTCGAGAAA		2
oqxAB	oqxAB-F	GTCCAGCGATAATCAGGC	669	3
	oqxAB-R	GGTCTCGGCAATCACTTT		3
floR	floR-F	CTGAGGGTGTCGTCATCTAC	673	4
	floR-R	GCTCCGACAATGCTGACTAT		4
rmtB	rmtB-F	ACATCAACGATGCCCTCAC	725	5
	rmtB-R	AAGTTCTGTTCCGATGGTC		5
gyrA	gyrA-F	ATGAGCGACCTTGCGAGAGAAATTACACCG	630	6
	gyrA-R	TTCCATCAGCCCTTCAATGCTGATGTCTTC		6
parC	parC-F	ATGAGCGATATGGCAGAGCG	589	6
	parC-R	CGATTAATGCGATTGCCGCCTGA		6
16S rRNA	16S-F	AGAGTTTGATCCTGGCTCAG	1465	7
	16S-R	GGCTACCTTGTTACGACTT		7

References

1 Liu JH, Wei SY, Ma JY *et al*. Detection and characterization of CTX-M and CMY-2 beta-lactamases among *Escherichia coli* isolates from farm animals in Guangdong Province of China. *Int J Antimicrob Agents* 2007; **29**: 576-81.

2 Hou J, Huang X, Deng Y *et al*. Dissemination of the fosfomycin resistance gene *fosA3* with CTX-M β-lactamase genes and *rmtB* carried on IncFII plasmids among *Escherichia coli* isolates from pets in China. *Antimicrob Agents Chemother* 2012; **56**: 2135-8.

3 Wang J, Guo ZW, Zhi CP *et al*. Impact of plasmid-borne *oqxAB* on the development of fluoroquinolone

resistance and bacterial fitness in *Escherichia coli*. *J Antimicrob Chemother* 2017; **72**: 1293-302.

4 Chen S, Zhao S, White DG *et al*. Characterization of multiple-antimicrobial-resistant *salmonella* serovars isolated from retail meats. *Appl Environ Microbiol* 2004; **70**:1-7.

5 Chen L, Chen ZL, Liu JH *et al*. Emergence of RmtB methylase-producing Escherichia coli and *Enterobacter cloacae* isolates from pigs in China. *J Antimicrob Chemother* 2007; **59**: 880-5.

6 Ahmed AM, Miyoshi S, Shinoda S *et al*. Molecular characterization of a multidrug-resistant strain of enteroinvasive *Escherichia coli* O164 isolated in Japan. *J Med Microbiol* 2005; **54**: 273-8.

7 Kim TW, Kim YH, Kim SE *et al*. Identification and distribution of *Bacillus* species in *doenjang* by whole-cell protein patterns and 16S rRNA gene sequence analysis. *J Microbiol Biotechnol* 2010; **20**:1210-4.

Table S2 Primers used to determine genetic environment of *mcr-1*

Region [a]	Nucleotide sequence (5'-3')	Size (bp)
IS*Apl1*-*pap2*	F: TCAACTTCGCTCCACT	1346
	R: GACTTTGATCCTAGTGCC	
pap2-*mcr-1*	F: CACCACCGTATGGCTCA	1487
	R: TCTGTGCCGTGTATGTT	
mcr-1-IS*Apl1*	F: CAGATGGCGTTGTTGGT	1692
	R: ACTGGGTAAAGCGGGTG	

Table S3 Primers used to assemble plasmid pHN6DS2

Region	Nucleotide sequence (5'-3')	Size (bp)	Position
HP1-IS*26*-*aphA1*	F: CAAGGGCAATCTGGTAT	1535	contig 1
	R: GTATTTCGTCTCGCTCA		contig 9
aphA1-IS*26*-r*epN*	F:CCTGATTGCCCGACATTA	1355	contig 9
	R:CGATTTCCCAGATACCAC		contig 8
gshB-IS*1294*-IS*26*-*mphA*	F:GGTGTTGGTGTCAGGGTA	1453	contig 8
	R:AACGGCAGGCGATTCTTG		contig 5
IS*6100*-IS*26*-Tn_{chrA}	F:TAGCTGATCGGATAGCG	1238	contig 5

	R:AACGGCGTGTTTATGAC		contig 4
tetM-IS*26*-*mefB*	F:AATCCCTGCTCGGTGTA	1395	contig 4
	R:AGCCTCCCTATCTGTAA		contig 2
IS*1006*-IS*CR2*	F:CGCACAGCCCAAAGAAT	1590	contig 2
	R:GCAGGATGGCGAGAAGG		contig 7
IS*CR2*-*floR*-ΔISCR2-*sul2*	F:CATGCTGTTTCTCGACGGT	1589	contig 7
	R:CGCAGGTGGAGCTGACT		contig 3
aac(*3*)-*IV*-IS*26*-*bla*$_{\text{CTX-M-14}}$	F:GGCCTCGATCAGTCCAA	1265	contig 3
	R:CCATCTCAAACTCCCAATA		contig 6
fosA3-IS*26*-Tn*1721*	F: CAGGCGTCTGTTGTGGT	1447	contig 6
	R: CCGTCCTTCAAGTGGGT		contig 1

Table S4 Primers used to detect the presence of pHN6DS2-like plasmid

**Region**	**Nucleotide sequence (5'-3')**	**Position in pHN6DS2**	**Size (bp)**
HP1-IS*26*-*aphA1*	F: CAAGGGCAATCTGGTAT	74433-74449	1535
	R: GTATTTCGTCTCGCTCA	75951-75967	
IS*26*-*repN*	TCTCCTCCCGTCGTAA	76869-76884	747
	CCAGCCTCTGTTAGCC	77600-77615	
aphA1-IS*26*-r*epN*	F:CCTGATTGCCCGACATTA	76293-76310	1355
	R:CGATTTCCCAGATACCAC	77630-77647	
gshB-IS*1294*-IS*26*-*mphA*	F:GGTGTTGGTGTCAGGGTA	79971-79988	1453
	R:AACGGCAGGCGATTCTTG	81406-81423	
fosA3-IS*26*-Tn*1721*	F: CAGGCGTCTGTTGTGGT	126187-126203	1447
	R: CCGTCCTTCAAGTGGGT	127617-127633	
Tn*1721*-HP2	F: GCTATCGTGCTGTGGAA	128592-128608	552
	R: CGTATGCTTTCGTGTAA	129127-129143	
HP3-*pap2*	F: AACCTACCCTATCGTGC	176946-176962	954
	R: ATGCCAGTAGCGGATTT	177883-177899	
IS*Apl1*-HP4	F: ATGGTGCTGGAGCTACGT	181141-181158	1306
	R: TCTTTCCCTGTTCCGATT	182429-182446	

HP: hypothetical protein, located in IncHI2 plasmid backbone. The last two primers (HP3-*pap2* and IS*Apl1*-HP4) were aimed to detect the insertion site of the module (*pap2*-*mcr-1*-IS*Apl1*) at IncHI2 plasmid

Table S5 Frequency of conjugal transfer of Five *mcr-1*-bearing plasmids

Plasmid	Plasmid sizes and typing	Original strain	Mean conjugal frequency
pHN6DS2	~244.4 kb, IncN1-HI2/ST3	GZ6DS2	2.67×10^{-3}
pHN6DS9	~244.4 kb, IncN1-HI2/ST3	GZ6DS9	4.67×10^{-3}
pHN6DH17	~244.4 kb, IncN1-HI2/ST3	GZ6DH17	3.3×10^{-3}
pHN6DH18	~244.4 kb, IncN1-HI2/ST3	GZ6DH18	5.0×10^{-2}
pHN6DS4	~70 kb, IncI2	GZ6DS4	7.22×10^{-4}

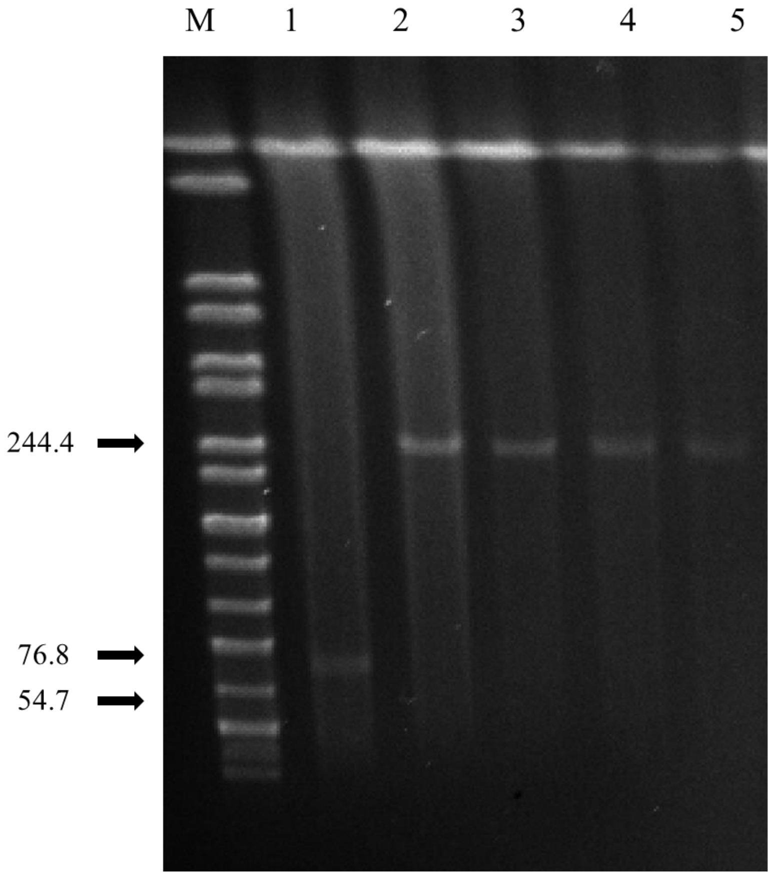

Figure S1 S1-PFGE of five *mcr-1*transconjugants. Transconjugants: Lane 1 GZ6DS4F-5C; 2 GZ6DS2-1C; 3 GZ6DS9-2C; 4 GZ6DH17-3C; 5 GZ6DH18-1C; M *Salmonella enterica* serovar Braenderup H9812 Marker.

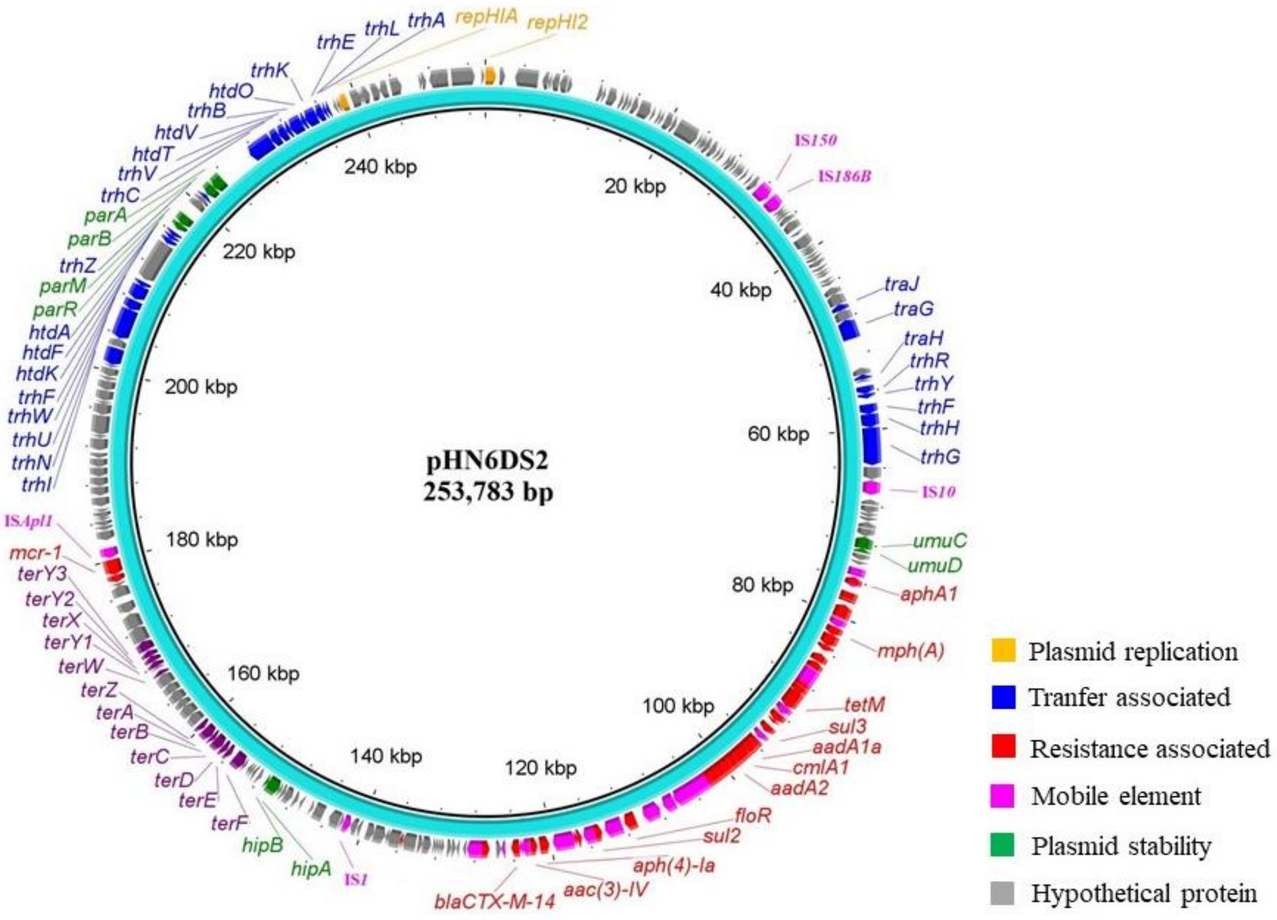

Figure S2 Circular map of plasmid pHN6DS2. Arrows indicate positions and direction of transcription of genes.
